# Composites of Graphene and LiFePO_4_ as Cathode Materials for Lithium-Ion Battery: A Mini-review

**DOI:** 10.1007/s40820-014-0004-6

**Published:** 2014-09-27

**Authors:** Haixia Wu, Qinjiao Liu, Shouwu Guo

**Affiliations:** Key Laboratory for Thin Film and Microfabrication of the Ministry of Education, School of Electronic Information and Electrical Engineering, Shanghai Jiao Tong University, Shanghai, 200240 People’s Republic of China

**Keywords:** Lithium iron phosphate, Graphene, Composite, Electrochemical property, Lithium-ion battery

## Abstract

This mini-review highlights selectively the recent research progress in the composites of LiFePO_4_ and graphene. In particularly, the different fabrication protocols, and the electrochemical performance of the composites are summarized in detail. The structural and morphology characters of graphene sheets that may affect the property of the composites are discussed briefly. The possible ongoing researches in area are speculated upon.

## Introduction

Environmental pollution and energy crisis have been severely accumulated due to the excessive utilization of fossil fuel resource. To overcome these problems, novel clear energy resource and related energy conversion and storage materials and devices are highly demanded. Among the diverse energy storage devices, lithium-ion batteries (LIBs) have been studied overwhelmingly, and certain kinds of LIBs have been commercialized already. LiCoO_2_ is one of the main LIB cathode materials used in industrial scale, but LiCoO_2_ could pollute the environment during production process, overcharge during usage thus causing potential safety hazard, and cobalt is expensive for its limited storage [1]. Therefore, looking for alternative materials of LiCoO_2_ is always the research hotspot. With the similar crystal structure as LiCoO_2_, LiNiO_2_ has an advantage of lower price, but there are difficulties in the synthesis, poor structure, thermal, and cycling stability [2]. Spinel LiMn_2_O_4_ has good security, ease of synthesis, etc., however, because of the presence of John–Teller effect in lattice during the charge/discharge, its structure is prone to distort, resulting in the rapid decay of the capacity, especially at higher temperatures [3]. Therefore, the exploits of high-performance electrode materials, electrolytes, and membrane for LIBs have attracted great attention during last decades [4]. Lithium iron phosphate (LiFePO_4_, LFP) with olivine structure is one of the most promising cathode materials for LIBs, owing to its high theoretical capacity (170 mAh g^−1^), acceptable operating voltage (3.4 V vs. Li^+^/Li), good cycling stability, low toxicity, good thermal stability, and low cost. And the biggest advantage of LFP is non-toxic compared to LiMPO_4_ (M = Co, Mn, and Ni) [5]. Unfortunately, the LFP shows intrinsically poor electrical conductivity (about 10^−9^–10^−10 ^S cm^−1^) and low Li^+^ transport capability (approximately 10^−14 ^cm^2 ^s^−1^) [6, 7], which constrains its electrochemical performance, especially the rate capability, as cathode in LIBs [4]. So far, numerous attempts have been made to speed up the Li diffusion within LFP crystals and to increase its electrical conductance by doping the LFP with other metal ions [8], reduction LFP particle size [9], coating conductive carbon layer [10–13] and aliovalent doping [8, 14–16]. It was demonstrated that the metal ion doping is not only able to expand the Li^+^ diffusion channel, but also increase the output voltage of LFP. The reduction of LFP particle size can shorten the Li^+^ diffusion path, but the disadvantage is to introduce the interface effect [17]. The carbon coating has been successfully used to improve the electrical conductivity of LFP crystals, though it may lower the energy density of the LIBs [4]. Additionally, the carbon coating can be easily accomplished through an in situ pyrolysis of organic carbon precursors, such as sucrose [18], glucose [19], starch [20], citric acid [21–23], ascorbic acid [24], adipic acid [25], pitch carbon [18], polypropylene [24], polypyrrole [26], polyvinyl alcohol [13], polythiophene [27], and polyacene [28] on the LFP. However, the composition, graphitization extent, thickness, surface functionality, and uniformity of the carbon coating layer are hard to be controlled in practice, which, on the other hand, affect significantly the electrochemical performance of LIBs in practice [29–31].

Having high carrier (ion and electron) mobility, good mechanical, and chemical/physical properties, graphene and derivatives have shown great potential in LIBs, and there are a large number of the research works related to the area so far [32, 33]. In this mini-review, we summarize the recent progress in studies on the LFP/graphene composites that is considered as one of the most promised cathode materials for high-performance LIBs. We first overview the synthetic protocols of the composites developed so far. Then, the structural and morphology characters of graphene sheets that may affect the property of the composites are discussed briefly. Finally, the possible ongoing developments and challenges in this area are speculated upon.

## LFP/Graphene Composites Prepared Through Physical Mixing

**Fig. 1:**
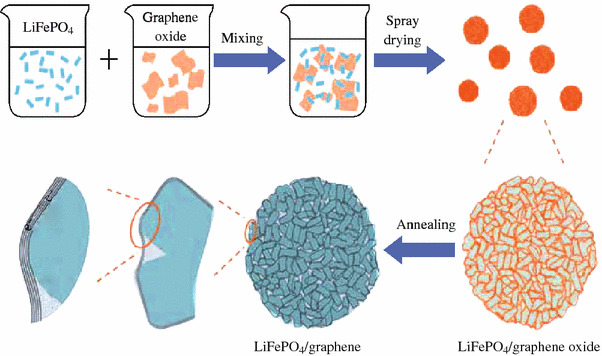
Schematic illustration of the preparation process and the proposed microstructure of LFP/graphene composite [48]

The crystalline LFP particles used in LIB cathode can be routinely prepared through sol–gel, hydrothermal, or solid-state reactions using different precursors [34–36]. The graphene sheets can usually be generated through mechanical exfoliation of the bulk graphite [37], chemical vapor deposition, CVD [38–40], chemical reduction of graphene oxide, GO [41–44], and electrochemical synthesis [45–47]. For the bulk scale preparation of the individual graphene sheets, the chemical reduction of the GO is often used [41–43]. To obtain the LFP/graphene composites, there has been much work toward the simple physical mixing of the LFP particles and graphene sheets. For example, using LFP nanoparticles and chemically reduced GO (rGO) sheets as raw materials, Zhou et al. developed a facile procedure, including the physically mixing of LFP and rGO suspensions to generate the slurry with certain LFP–rGO ratio, the spray-drying the slurry, and finally the thermal annealing in Ar (Fig. 1) [48, 49]. It was demonstrated that in the as-prepared composites the LFP nanoparticles were coated (actually wrapped) homogeneously with rGO sheets forming three-dimensional (3D) network, a favorable structural motif for facilitating the electron and lithium-ion migration throughout the composites. The LIB cathode prepared with the as-generated composite showed a specific capacity of 70 mAh g^−1^ at 60 C discharge rate and exhibited a capacity decay rate of <15 % when cycled at 10 C charging and 20 C discharging rate for 1,000 cycles [48]. Considering simple and scalable advantages, this strategy may be developed into a general way to prepare other graphene based composites for LIB cathodes, such as the composites of LiCoO_2_/graphene, LiMn_2_O_4_/graphene.

**Fig. 2:**
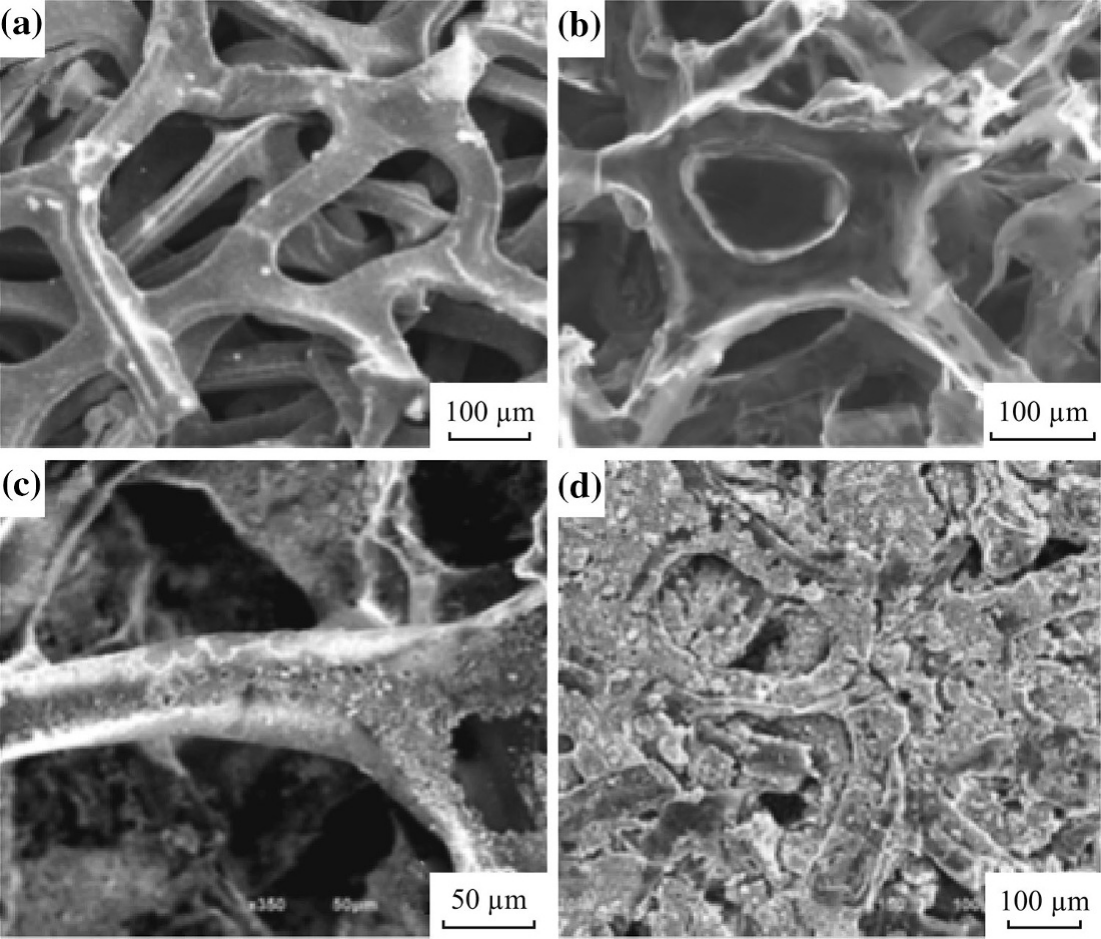
SEM images of **a** the Ni foam template, **b** 3D graphene network, **c** 3D graphene/LiFePO_4_ composite, and **d** the electrode surface of 3D graphene/LiFePO_4_ [50]

Although the rGO sheets can be readily prepared in bulk scale, its low electrical conductivity is a drawback for modification of the LFP cathode for LIBs. Therefore, Tang et al. [50] prepared first the multilayered graphene (MLG) with 3D network structure using commercially available porous Ni as template, and then intercalated the LFP nanoparticles within the 3D graphene to get finally graphene/LFP composites (Fig. 2). The high electrical conductivity (∼600 S cm^−1^) and unique structure of the as-prepared 3D graphene afford the composite with good electric and electrochemical properties. In contrast to the pure LFP, the cathode made of the 3D graphene/LFP composites showed higher specific capacity. The specific discharge capacities can reach to 158, 150, 144, and 135 mAh g^−1^ at discharging rate of 0.2, 1, 2, and 5 C, respectively, which are much higher than those of the pure LFP. Even at the rate of 10 C, the specific discharge capacity still remains at 109 mAh g^−1^, revealing the improved rate performance of 3D graphene/LFP, too. However, the laborious and costly preparation procedure may limit the practical application of such kind of the composite.

**Fig. 3:**
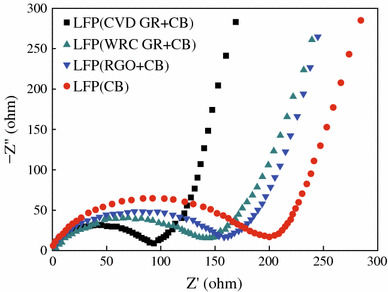
Electrochemical impedance spectra of LFP (CVD GR + CB), LFP (WRC GR + CB), LFP (rGO + CB) and LFP (CB) cathodes [56]

The graphene and rGO modifications can improve somehow the electrochemical performances of LFP, especially the rate capability, but the overall properties of the LFP/graphene composites seem depend strongly on the preparation procedures, and also the raw materials used [51–55]. To get insight into the effects of the graphene on the electrical and electrochemical properties of the LFP/graphene composites, Bi and colleagues prepared three kinds of the graphene sheets through CVD, wurtzite-type reductive coupling (WRC), and chemical reduction of the GO (rGO), and prepared the composites of LFP with the as-prepared different graphene sheets [56]. For comparison, they prepared also carbon black (CB)-coated LFP with the carbon layer thickness of 10–30 nm. It was demonstrated that the composite with CVD graphene showed an excellent electrical conductivity of 1,097 S cm^−1^, which is much larger than those with WRC graphene (3.0 S cm^−1^), rGO (1.2 S cm^−1^), and CB (0.5 S cm^−1^). In addition, electrochemical impedance spectroscopy measurement revealed, as shown in Fig. 3, the resistance of the cathode composed of LFP with CVD graphene is about 92 Ω, which is much smaller than those of LFP with WRC graphene (142 Ω), LFP with rGO (161 Ω), and LFP coated with CB (199 Ω). Accordingly, the CVD graphene-modified LFP cathodes exhibit larger reversible capacities of 132 and 80 mAh g^−1^ at even high charge/discharge rates of 1 and 20 C [56]. The reason might be that the CVD graphene has better interface contacting with active materials (LFP), resulting in better electrical conductivity and enhanced charge transfer.

## Growing LFP Particles In Situ on Graphene

As aforementioned, through simple physical mixing, the LFP/graphene composites can be prepared routinely, and the electrical and electrochemical properties of the composites as cathode for LIBs could also be improved somehow. However, their rate capability and cycling stability seem not significantly enhanced. The reason might be that the graphene sheets contact the LFP particles loosely through weak physical interactions. Therefore, if the LFP particles were tightly anchored onto or entirely encapsulated into the pristine graphene sheets, the electrical and electrochemical properties of LFP should be greatly enhanced. Fortunately, several procedures have been proposed for growing the LFP particles in situ on the surface of graphene [14, 57–60].

For example, through a high temperature solid chemical reaction, Wang et al., created LFP nanoparticles in situ on the surface of rGO sheets [57]. It was found that the LFP nanoparticles randomly distributed on the rGO sheets with the average size of 200 nm. The composites exhibited an initial discharge capacity of 161 mAh g^−1^ at 0.1 C and the capacity retained 70 mAh g^−1^ even at high rate of 50 C [57]. Similarly, Xu et al. [14] prepared graphene-encapsulated LFP nanospheres by a solid-state reaction using GO-encapsulated FeOOH, LiCH_3_COO·2H_2_O, NH_4_H_2_PO_4_ (in molar ratio = 1:1:1) as raw materials. They showed the LFP nanospheres (~20 nm in diameter) were wrapped tightly with a 3D graphene network. The as-prepared graphene-encapsulated LFP showed decent specific capacities of 166.6, 108.6, and 90.6 mAh g^−1^ at 0.1, 5, and 10 C, respectively, and the capacity decay can maintain at <9 % when cycled at 5 and 10 C charge/discharge rates for 300 times.

LFP can also be grown in situ on graphene sheets through wet chemical approaches. For instance, Wang et al. [58] prepared first the suspension containing LiOH, FeSO_4_·7H_2_O, H_3_PO_4_, ascorbic acid, and GO, in which the molar ratio of Li:Fe:P was adjusted to 3:1:1, and the weight ratio of GO to LFP was 8:92. The mixture was then transferred into a Teflon-lined stainless steel autoclave and heated at 200 °C for 5 h. The LFP nanoparticles were grown on the graphene sheets, and the as-prepared LFP/rGO composite as cathode exhibited also excellent electrochemical performances with capacities of 160.3 and 81.5 mAh g^−1^ at 0.1 and 10 C rates, respectively. A similar protocol was also developed by Ding et al. [60].

**Fig. 4:**
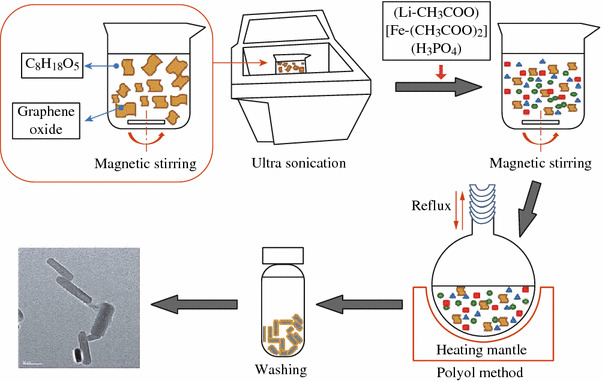
Schematic illustration of typical procedures used to prepare graphene-wrapped LFP nanorod [59]

In steading of the aqueous solution, Oh et al. [59] developed a low temperature polyol method using tetraethylene glycol [HO(CH_2_CH_2_O)_3_CH_2_CH_2_OH] as solvent to grow the LFP particles on graphene and the procedure is schematically illustrated in Fig. 4. In comparison, the LFP particles as-generated have unique nanorod-like morphology. The unique nanorod morphology, moderate size distribution, and uniform graphene coating enhanced the electrical and electrochemical properties tremendously. The specific capacities can reach up to 164, 156.7, and 121.5 mAh g^−1^ at current rates of 0.1, 1, and 10 C, respectively, with capacity retention ratios over 99 % after 100 cycles.

**Fig. 5:**
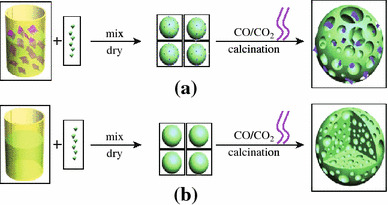
Formation process of the 3D porous networks for **a** LFP/graphene and **b** LFP [61]

**Fig. 6:**
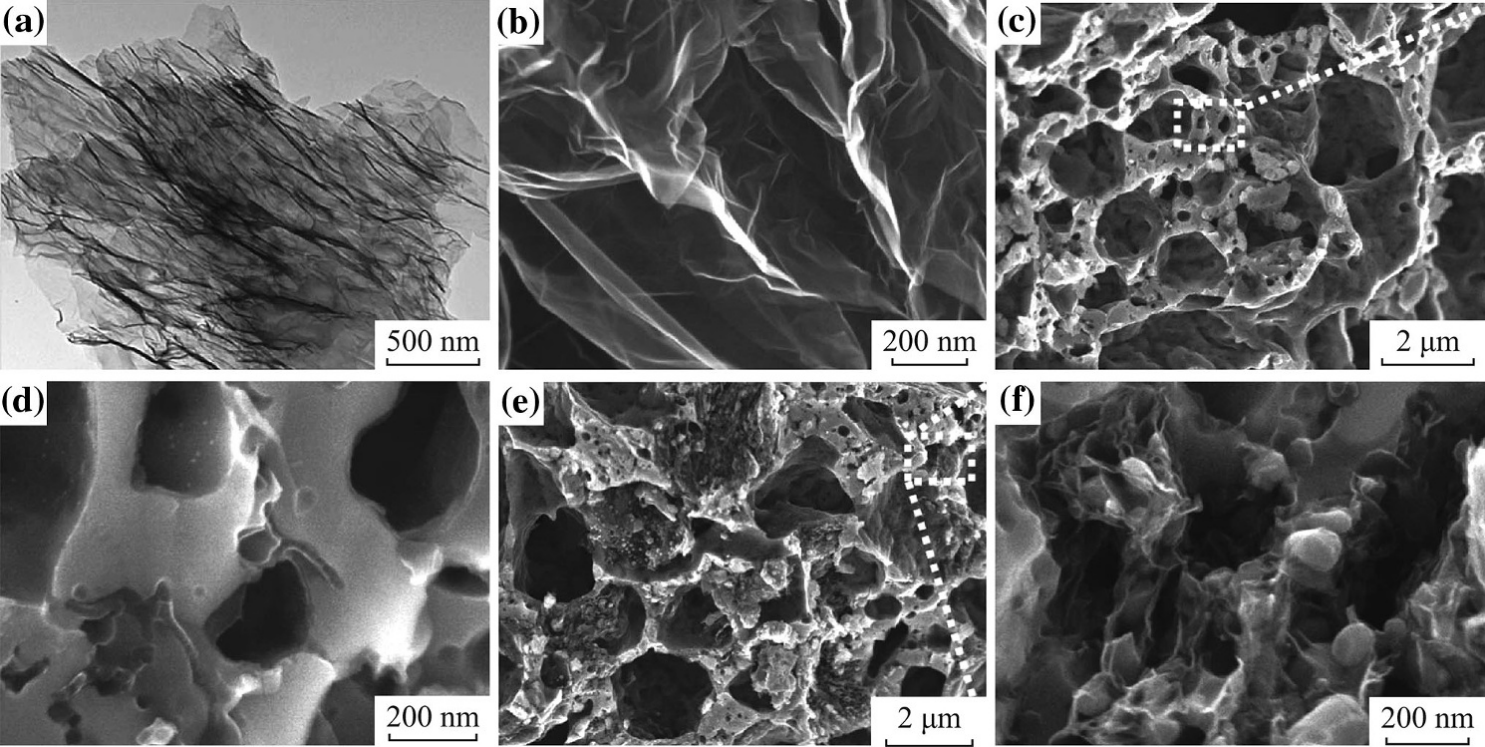
**a** TEM image of graphene. **b** SEM images of graphene. **c**, **d** Porous LFP at different magnifications. **e**, **f** LFP/graphene composite at different magnifications [61]

Significantly, the LFP/graphene composites assuming more complicated 3D hierarchical structure have been prepared through a facile template-free sol–gel procedure, as shown in Fig. 5 [61]. The approach for LFP/G composite, schematically illustrated in Fig. 5a, starts with the dispersion of graphene sheets in deionized water, proceeds with the self-assembly of graphene with the LFP precursor, and ends with the crystallization of the LFP/G precursors. CO and CO_2_ were evolved from the degradation of these precursors through annealing, resulting in the formation of a porous 3D network to obtain the final LFP/G product. A 3D cross-sectional view of LFP formation is shown in Fig. 5b. The high resolution scanning electron microscopy (SEM) and transmission electron microscopy (TEM) illustrated that the graphene sheets were dispersed uniformly into the pores of LFP (see Fig. 6). In comparison with porous LFP, the LFP/graphene composite shows significantly enhanced Li-ion insertion/extraction kinetics. More generally, the as-developed method seems applicable to other graphene-based composite material fabrication.

## Growing Graphene In Situ on LFP

As mentioned above, no matter using the physical mixing of LFP with graphene sheets or the in situ growing of the LFP on graphene sheets, the preparation of individual graphene sheets is prerequisite. Additionally, the individual graphene sheets usually aggregate easily, which affects the distribution of the graphene sheets within the composites that influence finally the electrochemical performance of the composites [62, 63]. To overcome the shortcomings, a bottom-up strategy was designed to grow in situ the graphene sheets on LFP surface. It was reported that using the dodecylamine bilayers deposited on the LFP as carbon source the MLG sheets were generated in situ on the LFP surface under high temperature graphitizing in inert atmosphere [64]. It is worth pointing out that the formation of graphene layers seems catalyzed by the Fe species from LFP at high temperature. The experimental data also demonstrated that such a precisely designed LFP/graphene composite shows a strikingly high electrical conductivity of 18.9 S cm^−1^ (in comparison with 10^−9 ^S cm^−1^ of pure LFP), a high lithium-ion capacity of 168 mAh g^−1^ (very close to its theoretical capacity of 170 mAh g^−1^ of LFP) at the 0.5 C rate, and a high rate capacity of 115 mAh g^−1^ at the 10 C rate. Moreover, the as-prepared LFP/graphene composite shows high cycling stability over the composites prepared through other methods.

**Fig. 7:**
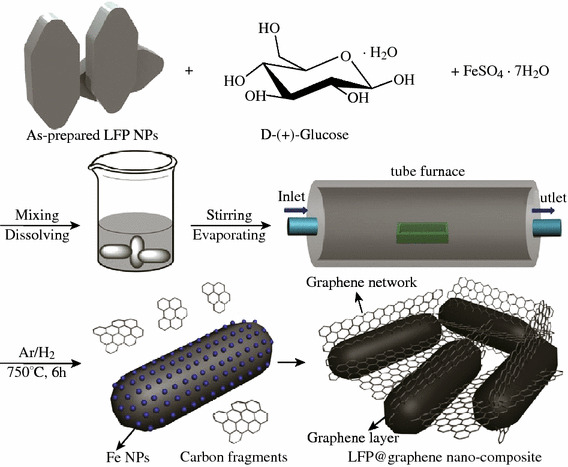
A schematic drawing (not to scale) of the overall preparation of the LFP/graphene composite through an in situ growing of graphene on the LFP reaction [31]

**Fig. 8:**
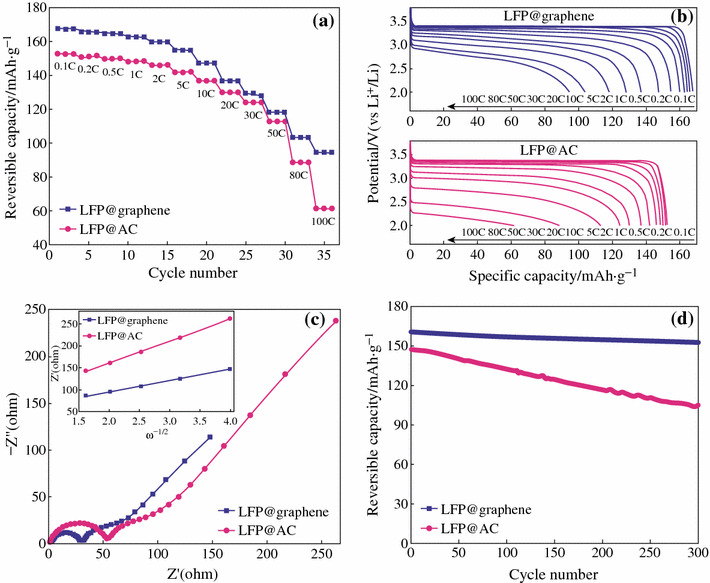
**a** Rate performance of the LFP@Active Carbon (AC) and LFP@graphene electrodes. **b** Discharge curves of the LFP@AC and LFP@graphene electrodes at various discharge rate. **c** Nyquist plots of the LFP@AC and LFP@graphene electrodes at 40 % DOD (depth of discharge) and plots of *Z*′ versus the square root of frequency (the *inset*). **d** Plots of reversible discharge capacity versus cycle number for the LFP@AC and LFP@graphene electrodes [31]

Similarly, using glucose as carbon source and FeSO_4_ as catalyst, Li et al. [31] prepared graphene on LFP through in situ catalytic pyrolysis and graphitization under Ar/H_2_ (95:5) at 750 °C, see Fig. 7. It was demonstrated that the as-grown graphene sheets has an average thickness of about 2.5 nm (about eight–nine layers). The graphene sheets not only coat uniformly the LFP surface, but also bridge the adjacent LFP particles together forming 3D network. Owing to the unique structure, as depicted in Fig. 7, the as-prepared LFP/graphene composite exhibited even better electrochemical properties. The reversible capacity is of 167.7 mAh g^−1^, and shows high rate performance and prolonged cycling life as shown in Fig. 8.

## Effects of the Structural and Morphology of Graphene Sheets on the Electrical and Electrochemical Performances of LFP/Graphene Composites

**Fig. 9:**
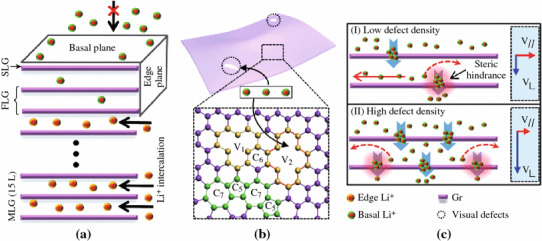
Lithium-ion diffusion in basal plane and edge plane [67]

It is well known that the graphene is a typical anisotropic material. The electron and lithium-ion transportations along the longitudinal and horizontal (in plane) directions should be different [65, 66]. Thus, the orientation of the graphene sheets on the LFP surface may severely affect the electrochemical performance of the composites as cathode. Actually, it was reported that the Li^+^ diffusion through a defect-free perfect graphitic plane is rather limited [67]. However, the LFP coated with graphene sheets exhibit usually the enhanced electrochemical properties as cathode in LIBs. To explain the doubt, Takamura et al. [68] proposed that there should be nanoholes in the graphene layers allowing Li^+^ to be very easily inserted and extracted via the holes, which was experimentally verified by the HRTEM analysis of the graphene used. To get insight the lithium diffusion pathway through the basal plane of graphene layers and the influence of defect population, Yao et al. [67] prepared monolayer graphene and MLG, respectively. Combining the experimental data and density functional theory calculations, they showed that basal plane hindered lithium-ion diffusion with a high diffusion barrier height, whereas vacancies and defects can be shortcuts for lithium-ion diffusion as shown in Fig. 9.

Actually, the lateral size of graphene sheets is also a key factor influencing the electrochemical performance of LFP/graphene composite as cathode in LIBs. For instance, Yang and colleagues [65] showed that instead of graphene sheets with micrometer lateral sizes, if the graphene nanosheets (GNRs) is used as additives, the as-obtained LFP/GNRs composites showed even better electrical and electrochemical properties than LFP/graphene. The reason might be that the GNRs bridge the LFP particles together forming more effective conductive networks, and the reduced lateral sizes lower the steric hindrance effect for the Li^+^ diffusion through the planar structure of graphene. This indicates additionally that the steric hindrance from the coating layer on the LFP for ion diffusion should be considered in further design of the surface modification of LFP.

Besides the orientation and the lateral size, the edge structure and thickness of the graphene sheets can also affect the property of LFP. Uthaisar and Barone [69] demonstrated theoretically that the armchair and zigzag edges appeared on the GNRs can affect the adsorption and diffusion capabilities of the Li atoms. The adsorbed Li atoms can diffuse toward the edges where the energy barrier is lower than within the graphene plane, which may increase significantly the diffusion coefficiency of Li atoms. The overall results showed that electrodes fabricated with GNRs should increase the power of Li-ion batteries. Lee and Persson [70] studied the mechanism and strength of Li absorption in graphene with different thickness (layer numbers) using so called cluster expansion method and the density functional theory calculations. It was predicted that the number of layer and also the defects of the graphene may play key roles in their Li storage. This assumption was proved later by Liu et al. [71].

Although the graphene sheets can improve the electrical and electrochemical performance of LFP as cathode in LIBs, however, it is hard to control specifically on the orientation, the size, and the edge structure in practice. Therefore, many researches were explored to using the synergistic effects of graphene and other carbon materials to increase the performances of LFP cathode materials [63, 72–74]. For example, Hu et al. [17] reported that the specific capacity of carbon-coated LFP modified with 2 wt% of the electrochemically exfoliated graphene layers is able to reach 208 mAh g^−1^. Through testing the coin cells with the cathodes made by 0 and 1.8 wt% graphene flakes on silica particles, they disclosed that the excess capacity is attributed to the reversible reduction–oxidation reaction between the lithium ions of the electrolyte and the exfoliated graphene flakes, where the graphene flakes exhibit a capacity higher than 2,000 mAh g^−1^. The highly conductive graphene flakes wrapping around carbon-coated LFP also assist the electron migration during the charge/discharge processes, diminishing the irreversible capacity at the first cycle and leading to ~100 % Columbic efficiency without fading.

## Summary and Outlook

In summary, we have reviewed the recent progresses in the studies on the composites of LFP/graphene. As one of the most promising cathode materials for LIBs, LFP has been exploited overwhelmingly; however, the poor electrical conductivity limited deadly the application of bare LFP in the LIB cathode. Graphene, a novel 2D material, has unique morphology and incomparable electrical conductivity and other attractive properties. It has been illustrated that the graphene sheets could be coated on the LFP surface through several controllable manners, including the simple physical mixing, in situ growth of the graphene onto LFP, and, alternatively, the in situ growth of the LFP onto graphene sheets. After the formation of the composites of LFP/graphene, the electrical and electrochemical performances of the LFP can be improved significantly. The structural characters of graphene sheets that may affect the properties of the composites have also been elucidated. The composites were found applicable as cathode for LIBs.

However, for the practical application of LFP/graphene composite, several fundamental issues remain to be solved and some possible solutions are as follow: first, the graphene sheets used so far for preparation of LFP/graphene composites were generated usually through the chemical reduction of the GO in which there are various oxygen containing groups, thus, the effects of the surface functionality of the graphene sheets on the interaction between the graphene sheets and LFP and the influences on the properties needs to be studied. Second, a reasonable theoretical model should be developed to describe the electron and Li-ion transportation through the LFP/graphene, the structure and morphology changes of LFP/graphene during the charge/discharge should be studied systemically, that will help to understand the mechanism of the storage mechanism of the Li-ions or atoms, which may also help for further rationally designing and preparing the composites with desired properties for practical application. Third, the scalable preparation procedures of the composites with homogeneous composition and controlled morphology need to be developed, and liquid process is recommended.
